# Clinical Neuropathology image 5-2014: α-synuclein pathology in the ependyma in Parkinson’s disease 

**DOI:** 10.5414/NPP33328

**Published:** 2014-08-18

**Authors:** Gabor Kovacs

**Affiliations:** Institute of Neurology, Medical University of Vienna, Vienna, Austria

**Keywords:** α-synuclein, Parkinson’s disease, Lewy-related pathology

## Abstract

Not available.

α-synuclein pathology presenting as tiny dots, thin neurites, and larger amorphous deposits in the subependymal area, as well as tiny dots between ependymal cells in the brains of individuals with neuropathologically proven Parkinson`s disease (Braak stages 5 and 6). Immunostaining was performed using antibody 5G4 (Roboscreen, Leipzig, Germany; we applied 1 : 2,000 dilution and a pretreatment of 10 minutes microwaving in citrate buffer, pH 6, followed by 5 minute 80% formic acid treatment). We recently reported that this antibody has strong selectivity for β-sheet rich α-synuclein oligomers and that the ependymal α-synuclein immunoreactivity correlates well with Braak stages of Lewy-related pathology [1]. The antibody is suitable for detecting α-synuclein pathology even in tissue fixed for a very long time in formalin [2]. We report here that 5G4 antibody is able to detect disease-associated α-synuclein in the cerebrospinal fluid of individuals with neuropathologically proven α-synuclein deposition in the brain [3]. 

**Figure 1:**
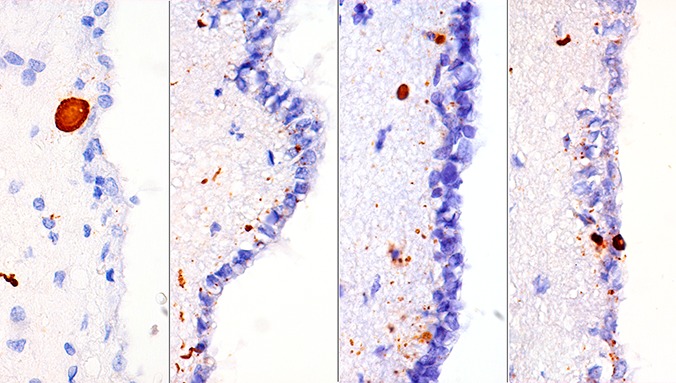
Clinical Neuropathology Image 5-2014: α-synuclein pathology in the ependyma.
